# Co-design, implementation, and evaluation of an expanded train-the-trainer strategy to support the sustainability of evidence-based practice guides for registered nurses and social workers in primary care clinics: a developmental evaluation protocol

**DOI:** 10.1186/s12875-022-01684-0

**Published:** 2022-04-18

**Authors:** Marie-Eve Poitras, Yves Couturier, Emmanuelle Doucet, Vanessa T. Vaillancourt, Marie-Dominique Poirier, Gilles Gauthier, Catherine Hudon, Nathalie Delli-Colli, Dominique Gagnon, Emmanuelle Careau, Arnaud Duhoux, Isabelle Gaboury, Ali Ben Charif, Rachelle Ashcroft, Julia Lukewich, Aline Ramond-Roquin, Sylvie Massé

**Affiliations:** 1 Department of Family Medicine and Emergency Medicine, Faculty of Medicine and Health Sciences, Université de Sherbrooke, 305 Rue Saint Vallier, Chicoutimi, Saguenay, QC G7H 5H6 Canada; 2 Academic Research Chair on Optimal Professional Practice in Primary Care, Université de Sherbrooke, Saguenay, Canada; 3 Centre intégré universitaire de santé et de services sociaux du Saguenay-Lac-Saint-Jean, Saguenay, Canada; 4 School of Rehabilitation, Université de Sherbrooke, IUPLSSS, CIUSSSE-CHUS, Sherbrooke, Canada; 5 Department of Social Work, Université de Sherbrooke, Sherbrooke, Canada; 6 Université du Québec en Abitibi Témiscamingue, Val-d’Or, Canada; 7 Department of Rehabilitation, Faculty of Medicine, Université Laval, Québec, Canada; 8 Faculty of Nursing, Université de Montréal, Montréal, Canada; 9 Department of Family Medicine and Emergency Medicine, Université Laval, Université Laval, Québec, Canada; 10 Faculty of Social Work, University of Toronto, Toronto, Canada; 11 Faculty of Nursing, Memorial University of Newfoundland, St. John’s, Canada; 12 Department of General Practice and Laboratory of Ergonomics and Epidemiology in Occupational Health, University of Angers, PRES L’UNAM, Université d’Angers, Angers, France

**Keywords:** Primary healthcare, Train-the-trainer, Nurses, Social workers, Practice guides, Family medicine

## Abstract

**Background:**

The implementation of evidence-based innovations is incentivized as part of primary care reform in Canada. In the Province of Québec, it generated the creation of interprofessional care models involving registered nurses and social workers as members of primary care clinics. However, the scope of practice for these professionals remains variable and suboptimal. In 2019, expert committees co-designed and published two evidence-based practice guides, but no clear strategy has been identified to support their assimilation. This project’s goal is to support the implementation and deployment of practice guides for both social workers and registered nurses using a train-the-trainer educational intervention.

**Methods/design:**

This three-phase project is a developmental evaluation using a multiple case study design across 17 primary care clinics. It will involve trainers in healthcare centers, patients, registered nurses and social workers. The development and implementation of an expanded train-the-trainer strategy will be informed by a patient-oriented research approach, the Kirkpatrick learning model, and evidence-based practice guides. For each case and phase, the qualitative and quantitative data will be analyzed using a convergent design method and will be integrated through assimilation.

**Discussion:**

This educational intervention model will allow us to better understand the complex context of primary care clinics, involving different settings and services offered. This study protocol, based on reflective practice, patient-centered research and focused on the needs of the community in collaboration with partners and patients, may serve as an evidence based educational intervention model for further study in primary care.

## Background

It is widely recognized that the Canadian healthcare system is based on the principals of primary healthcare, [1] and that the performance indicators of the structure, process, and outcome dimensions should be followed closely [2]. In a 2012 report, the Canadian Foundation for Innovation stated that Canada falls significantly behind in primary care compared to other countries of the Commonwealth in terms of restricted access to care, poor integration and coordination of services, problematic interprofessional collaboration, and limited patient participation [1]. In order to improve the performance of primary care, services in Québec have undergone multiple changes over the past 15 years, such as the creation of primary care clinics (PCCs) [3, 4], some of which include an academic mission [5]. Initially, these medical clinics were composed of family physicians and registered nurses (RNs) [6]. Several years later, the Québec Ministry of Health and Social Services required the inclusion of social workers (SWs) and pharmacists in these clinics [7, 8]. Other professionals who have joined PCCs, such as registered dietitians and physiotherapists, offer multiple services to a registered clientele in collaboration with family physicians and RNs [3]. PCCs are co-directed by a head physician and a regional health organisation, with several hospitals, long-term care centers, and local community service centers under their administrative supervision, and working with mostly independent PCCs. It is clear that PCCs have been the subject of a modernization effort in the last decade, in order to improve interprofessional collaboration and integration of services [9]. The arrival of RNs [3] and, more recently, SWs, [7] in PCCs and the implementation of innovative practices has improved the integration of psychological, physical, and social care, as well as care for patients with complex needs [10]. Despite improvements and operational innovations that PCCs have gone through [11, 12], their success is not guaranteed, due in part to the practice variation of RNs and SWs across these settings.

In 2016, the Québec Ministry of Health and Social Services developed practice guides in collaboration with various experts to inform decision-makers and head physicians who manage PCCs, among others, about the expected practice standards for RNs and SWs [13]. Both guides are composed of three sections: 1) information regarding the operation of PCCs; 2) the expected role for SWs or RNs in PCCs, and 3) interdisciplinary collaboration in PCCs. These guides also inform nursing and social work directors, primary care program directors, managers, and physicians responsible for PCCs of the standard of practice expected of RNs and SWs working in these clinics. In September 2019, the Ministry disseminated these guides throughout the province of Québec. Each healthcare center is responsible for their dissemination in the PCCs, despite the fact that a national strategy is currently being developed to support healthcare centers at this stage. In order to optimize the assimilation of new practices in clinical settings, there was a need for a distribution and implementation strategy to be identified and offered to professionals [14, 15]. Therefore, the government of Québec, in partnership with the Strategy for Patient-Centered Research (SPOR) Support Unit of Québec and the Fonds de Recherche du Québec en Santé (FRQS), searched for a team that could develop, implement, and evaluate a strategy to support the dissemination of the guides through a peer-reviewed call for funding. Our team was selected and was given this mandate.

## Methods and design

### Aims

The project’s overall goal is to support the implementation and deployment of both SW and RN practice guides using an expanded train-the-trainer (TTT) educational intervention. The specific aims are to:

1. Co-design and implement a training and support program consistent with the advocated practice changes in both guides;
2. Collect information about the best ways to implement a training program from participating healthcare centers;
3. Describe the training program implementation process;
4. Evaluate the implementation process among trainers, trainees, decision-makers, managers, practitioners, and patients; and
5. Identify successful conditions for scaling up. In this paper, we follow the methodological approach outlined within the Standards for Reporting Implementation Studies (StaRI) guidelines.

### Developmental design and assessment

**Fig. 1:**
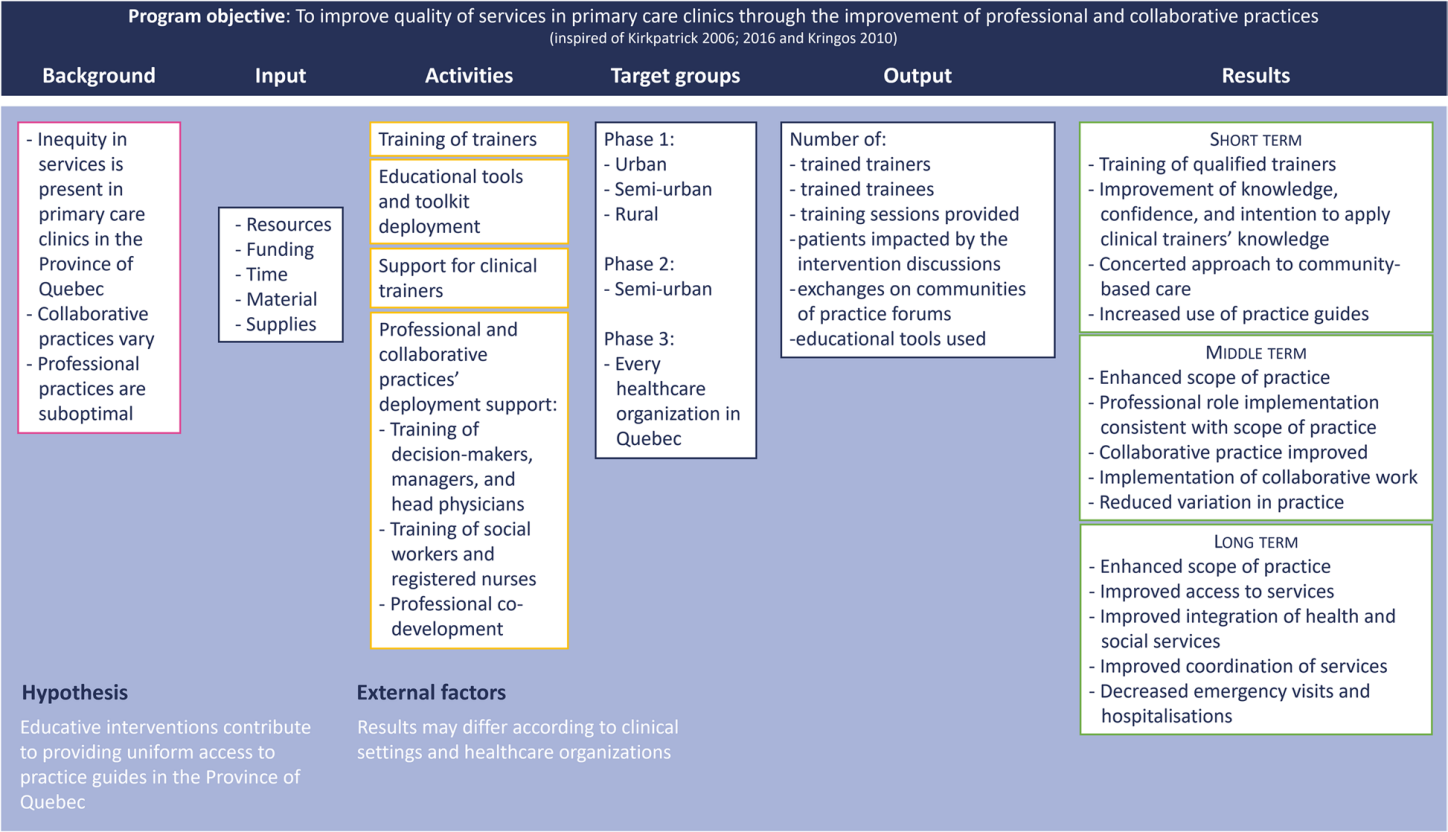
Presents the logic model and the relationships among the resources, activities, output, and outcomes

The proposed educational intervention will go far beyond the content of the training alone [16]. It will be based on the primary care monitoring system developed by Kringos [2]. This framework helps explain three dimensions of the primary care system: 1) structure, which includes governance, economic conditions, and workforce development; 2) process, which includes access to care, complementarity, continuity, and coordination of services; and 3) outcomes, which include quality, efficiency, and equity of care. This framework will allow us to establish links between governance and its various determinants, including adequate training of health and social care professionals as they contribute to improve the quality, efficiency, and equitability of primary care, when properly trained. We will also use the Kirkpatrick model [17] which aims to evaluate the efficiency of the TTT program in improving trainers’ learning and behaviors for training healthcare professionals (i.e., knowledge, attitudes, skills, competencies, commitment, and behavior). The logic model of our education intervention, informed by Kirkpatrick [17] and Kringos et al. [2], as well as evidence-based data regarding TTT interventions, are presented in Fig. 1.

**Fig. 2:**
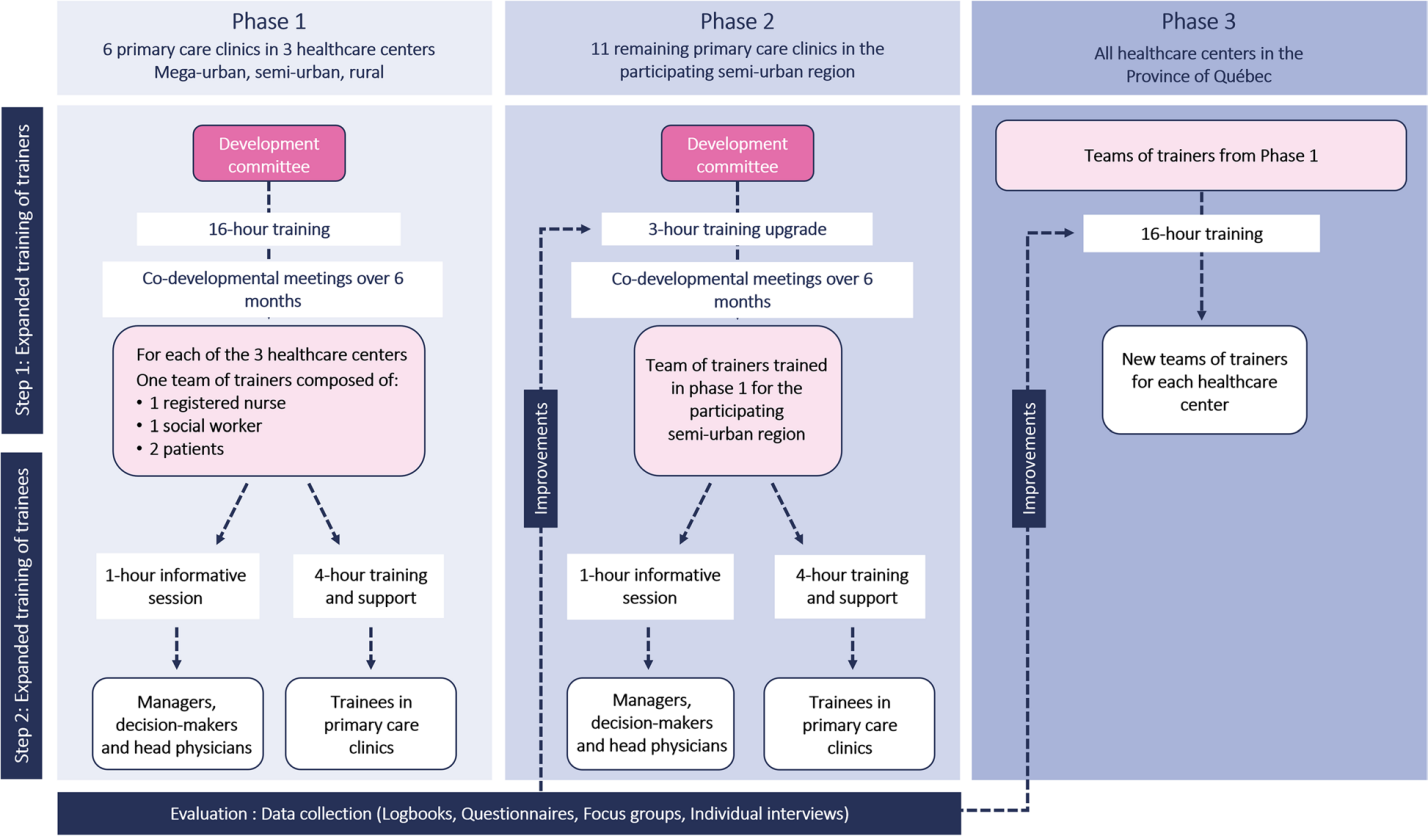
Shows the different phases and steps of the methodology

We will use a developmental assessment approach [18] to co-design and evaluate the TTT intervention. This project employs a developmental evaluation using a multiple case studies design with mixed data [19] in the form of a nested data collection [20], and will be carried out [19] with a before-and-after study methodology [21, 22]. In this study, a case is defined as a PCC. The developmental assessment approach [18] will allow us to fully understand the deployment of the educational intervention, the process of appropriating the content of the guides, the possible effects in the environments, and the indicators [23] to be considered for scaling up. It provides continuous information on the development and implementation of the educational intervention. After the co-design of the educational intervention, the project consists of three implementation phases grounded in an integrated knowledge translation approach [24]. These three phases are: 1) implementation and evaluation of the educational intervention in six PCCs in three administrative regions; 2) implementation and evaluation of the educational intervention in 11 PCCs in one administrative region; and 3) implementation of the educational intervention in each healthcare center of the province of Québec (evaluation of trainers only). Figure 2 summarizes the methodology.

### Co-design of the educational intervention

A development committee (Fig. 2) composed of clinician experts in knowledge transfer and the research team (researchers, patient partners, coordinator, clinician) will be formed to develop educational content. This committee will also ensure that educational content complies with the requirements of the community and is consistent with scientific evidence. The educational intervention will take place in two steps: 1) expanded training of teams of trainers; and 2) expanded training of trainees (RNs and SWs) in clinical settings. The co-design process will include multiple committee meetings to brainstorm about relevant content that must be included in the intervention and selection of interactive and appropriate andragogic strategies tailored for PCCs. The members of the committee will also perform individual reviews for each educational document. An iterative process will be coordinated by the research assistant to bonify the content and the andragogic strategies until each member is satisfied.

#### Step 1: expanded training of teams of trainers

The development committee will train the teams of trainers (Fig. 2,) with a 16-h training session. It will be composed of 10 themes in order to address the content of the practice guides but also to support trainers in the development of their training-related skills. The training program’s content will be available upon request after the project is complete. This significant expansion of training objectives will allow teams of trainers to develop their knowledge and skills as clinical trainers from an interprofessional collaboration perspective. With these new skills, they will be prepared to support practice changes beyond the content of the two targeted guides. The 10 themes related to PCCs will be:

1. The andragogy of the clinical trainer
2. Primary care, PCCs, and their position in care and service trajectories
3. Practice guides for RNs and SWs and their content
4. Scope of practice of RNs
5. Scope of practice of SWs
6. Interprofessional collaboration
7. Professional co-development
8. The elements that positively or negatively influence the deployment of professional and interprofessional practices and relevant coaching strategies
9. The care experience of people attending the PCCs
10. The suggested training sequence according to a macro-, meso-, and micro-approach

In addition to training, the educational intervention will include co-development meetings with the development committee over 6 months. These meetings will be held on a monthly basis (or according to a frequency established by the needs of the clinical teams) and will be in the form of professional co-development activities [25, 26]. These meetings will allow the teams of trainers to discuss barriers and facilitators to the deployment of their role as clinical trainers. A web platform will be created to allow them to exchange knowledge on several topics according to the clinical trainers’ emerging needs.

The teams of trainers will roll out in-practice strategies to support trainees in taking ownership of the content within the practice guides. The teams of trainers will carry out meetings with stakeholders according to the socio-institutional theoretical model of macro (decision-makers), meso (managers, head physician in PCC), and micro levels (clinicians) [27]. Each stakeholder identified by the healthcare center will be informed of their mandate and role, and will inform its team of trainers about the site’s priorities for local care and innovations that may be implemented through the practice guides. This support will help the team of trainers in deploying training and the expected practices according to the specific needs of the environments in which they work.

#### Step 2: expanded training of trainees (RNs and SWs) in clinical settings

The development committee will also co-design a four-hour training program for trainees of targeted settings. This program aims to present the guides, their content, and the ways in which clinicians view its assimilation in accordance with their PCC’s specific context. The themes will be:

1. Primary care, PCCs, and their position in care and service trajectories
2. Practice guides for RNs and SWs and their content
3. Scope of practice of RNs and scope of practice of SWs
4. Interprofessional collaboration
5. Professional co-development
6. The care experience of people attending the PCCs

Co-developmental meetings (one per month or more if needed) will be conducted by the teams of trainers. Frequency and modes of communication will be established during trainees’ training. Co-developmental meetings will allow for discussion of intraprofessional and interprofessional issues related to the assimilation of the guides throughout the 6 months of participation in the project. These meetings will contribute to building the credibility of trainees’ clinical activities, establishing a space for dialogue with members of the interprofessional team, and exercising their collaborative leadership while consolidating their scope of practice for the benefit of patients. Interprofessional case discussions will be conducted if trainees express the need. Members of the interprofessional team (e.g., physicians) of the PCC will be invited to the meetings to reinforce collaborative practices. The teams of trainers are considered an interprofessional team and will need to establish collaboration modalities among themselves and define methods from which they will support trainees. Individual or group coaching sessions will be offered by the team of trainers through this project and will not be a replication of clinical coaching activities already offered by the advisor in place. Briefly, these sessions will be offered over a period of 6 months and will aim to support trainees in putting into practice the content taught during the initial session. In order to do so, trainees will share clinical situations experienced in their work context and trainers will assist them in linking practice to theory by referring to practice guidelines and using a reflective approach, in a context of continual improvement. Coaching support will be adapted by the teams of trainers to the needs and unique situations reported.

### Evaluation and data collection

Following its development, the educational intervention will be implemented in an experimental phase in six PCCs located in three different healthcare centers (phase 1) (see sampling and recruitment method for selection criteria), then scaled up to all PCCs (*n* = 11) of the healthcare center of the participating semi-urban region (phase 2). The implementation will be evaluated throughout this phase, feeding further implementation continuously to conclude with a Québec-wide training strategy applied to all trainers in the province (phase 3).

The research team (coordinators, researchers, and patient partners) will collect qualitative data through logbooks, [28] focus groups, [28] and individual interviews to document: 1) training process and support in deployment; 2) patient healthcare experience; 3) implementation of the training program and the effects on trainers, decision-makers, and managers; and 4) assimilation process of new professional practices and interprofessional collaboration. This will inform the research team on the implementation of the educational intervention throughout the process, assimilation of the practice guides, effects observed, and conditions for scaling up. Quantitative evaluation will allow us to perceive possible effects and to identify the evolution of changes in practice in order to delve further into the qualitative evaluation process. Trainers and trainees will complete a validated [29] self-administered web questionnaire to describe learning related to the training received such as knowledge, confidence in applying learning, [30] and intention to apply knowledge [17, 31]. Sociodemographic data before training and reactions [32] following the training will also be documented. These surveys will inform the research team on: 1) learning from the TTT program offered by the research team; 2) learning from the training program offered to participants by the clinical trainers; 3) scope of practice and collaborative practice; and 4) sociodemographic data. Table 1 presents the variables under study, data sources, and measurement times for the three phases.

**Table 1:** Variables, data sources and measurement times

Data source		Measured and described concepts	References	Measurement time											
				Trainers’s training		Phase 1				Phase 2				Phase 3	
				Pre-	Post-	T0	T1	T2	T3	T4	T5	T6	T7	T8	T9
**Qualitative data**	Professional practice guides	Expected practice in primary care clinics	Ministère de la santé et des services sociaux. 2019 Guide pratique à l’intention des travailleurs sociaux issus d’un établissement du réseau de la santé et des services sociaux et qui travaillent dans un groupe de médecine de famille ou un groupe de médecine de famille universitaire. p. 41. Guide pratique à l’intention des infirmières cliniciennes qui travaillent dans un groupe de médecine de famille ou un groupe de médecine de famille universitaire. p. 76.			x				x					
	Logbooks	Training and support process deployment and activities carried out by clinical trainers and patient trainers	Guest, G., E.E. Namey and M.L. Mitchell, Collecting qualitative data: A field manual for applied research. 2013, Thousand Oaks, CA: Sage.	x	x	x	x	x	x	x	x	x	x	x	x
	Focus groups with patients	Experience with care and services				x		x	x	x		x	x		
	Focus groups with trainers, decision-makers, managers, physicians and continuous quality improvement agents	Implementation of the education intervention, its effects and process of assimilation of professional practices				x			x	x			x		
	Focus groups with social workers and registered nurses						x		x		x		x		
**Quantitative data**	Self-administered questionnaire on trainers’ learning	- Confidence in applying learnings Visual scale 0–10	Bandura, A., Guide for constructing self-efficacy scales. Self-efficacy beliefs of adolescents, 2006. 5 (1): p. 307–337	x	x									x	x
		- Intention to apply knowledge Analog visual scale 0–10	Kirkpatrick, J.D. and W.K. Kirkpatrick, Kirkpatrick’s Four Levels of Training Evaluation. 2016: Association for Talent Development	x	x									x	x
	Self-administered questionnaire on clinician’s learning	- Response to training and trainer performance 5 yes/no questions	Kirkpatrick, D.L., Implementing the Four Levels: A Practical Guide for Effective Evaluation of Training Programs. 2009: ReadHowYouWant.Com			x	x								
	Self-administered questionnaire on collaborative practices and scope of practice	- Collaborative practices 6 dimensions, 41 items Likert Scale 1–6	Careau E, Paré L, Maziade J and S. Dumont, CoPIP: Évaluation des compétences à la collaboration interprofessionelle (v1.2).2014			x	x	x	x	x	x	x	x		
		- Scope of nursing practice 26 activities, 6 dimensions, Likert scale 1–6	Braithwaite, S. (2016). *Measuring Scope of Practice Enactment among Primary Care Registered Nurses in Ontario* (Doctoral dissertation).			x	x	x	x	x	x	x	x		
		- Scope of social workers’ practice 75 activities, 4 dimensions Likert scale 1–6	Delli-Colli, N., N. Dubuc, R. Hébert and M.-F. Dubois, Measuring Social-Work Activities with Older People. Practice, 2013. 25 (5): p. 281–296.			x	x	x	x	x	x	x	x		
	Self-administered sociodemographic questionnaire	- Home-made questionnaire 10 items		x		x				x				x	

#### Phase 1 - selection and training of teams of trainers from three regions

Three healthcare centers were already targeted to be part of this project based on their area characteristics and include mega-urban, semi-urban, and rural classifications in three administrative regions of the province of Québec, Canada. This will allow us to understand how the education intervention should be deployed and adapted in each specific context. The managers of these participating sites will identify one RN, one SW and two patients partners to be trained and form a team of trainers. To be eligible to become a trainer, the RN and SW should hold a coordination or advisory role in their organization. They should be involved in the clinical support of primary care teams to ensure the sustainability of the coaching implemented beyond the project’s end. The team of trainers will receive the educational intervention and will train trainees in clinical settings. They will share tasks based on their availability and health status (mostly for patient partners). The measurement times for this phase will be pre – and post – teams of trainers’ training.

##### Selection and training of trainees from 6 clinical settings

Among the participating healthcare centers, using a non-probability sampling, we will target two PCCs [33] according to their sociodemographic and clinical characteristics. A minimum of two SWs and six RNs in each PCC will undergo a four-hour training session by their local team of trainers. Decision-makers, one manager, one physician, and one continuous quality improvement agent will also be included in the research process. Finally, 30 patients with conditions favouring interprofessional collaboration (loss of autonomy, multimorbidity, etc.) will also be involved in phase 1. For enrolled patients, inclusion criteria are as follows: 1) be registered in the participant PCC; 2) be 18 years of age or older; 3) live in the PCC area and not expect to leave this area for the duration of the project; 4) be able to provide informed consent; and 5) speak French. These patients will inform us on the impact of training from a patient’s point of view.

For phase 1, the data collection times will be: T0 - before stakeholder training; T1 - after stakeholder training; T2–1 month after stakeholder training; T3–6 months after stakeholder training; and T4–12 months after stakeholder training. Logbooks will also be filled by clinical trainers throughout the project to document the facilitators and barriers of the implementation.

#### Phase 2- training of trainees in all remaining semi-urban region clinical settings

The second phase of the research project will aim to scale up the education intervention of phase 2 to all PCCs of the semi-urban region (*n* = 11). The research team will use evidence from the previous phase to improve the intervention based on lessons learned throughout the study and the specific needs of each healthcare center. The same regional team of trainers involved in phase 1 will train an expected total of 11 SWs and 22 RNs to be trainees, as well as 55 patients-partners, six managers, six decision-makers and one continuous quality improvement agent as participants.

For phase 2, data will be collected: at T2–1 month after stakeholder training, at T4 – before the training of participants, at T5 – after the training of participants; at T6–1 month after training; at T7–6 months after training. An interview with the clinical trainers will also be done at T4 to describe their experience of training and coaching during phase 1.

#### Phase 3 - selection and training the teams of trainers from all remaining healthcare centers from the province of Québec

For this phase, the research team will propose to deploy an optimal educational intervention in every healthcare center of the province of Québec. Invitations will be extended to each center and they will appoint two clinical trainers (one SW and one RN) and two patient trainers to compose their own team of trainers. The teams of trainers from phases 1 and 2 will train these new teams of clinical trainers. For phase 3, the measurement times will be T8 – before and T9 – after provincial training of trainers.

### Data analysis

For each case (i.e., each PCC) and for each phase, qualitative data will be analyzed according to three concurrent streams: condensation (e.g., selection, transformation of raw data), presentation (e.g., narrative text, table, matrix) and verification of conclusions (e.g., go back to field notes, discussion with stakeholders) [34]. This method allows for an iterative process in which, for example, data coding (condensation) leads to the identification of new themes that need to be explored (presentation) and that may lead to new conclusions. The following themes will be explored: 1) facilitating factors and barriers related to the contexts and processes that may have influenced the deployment of the education intervention and the assimilation of the content of the practice guides; and 2) the effects of the education intervention on the clinical trainers, trainees, patients, decision-makers, and managers. From a developmental evaluation perspective, [18] data will be analyzed iteratively to inform the project team and clinical trainers’ real-time impacts, barriers, and facilitators. A specialist will transcribe interviews and focus group audio files verbatim and we will perform a qualitative deductive/inductive thematic analysis [35, 36]. We will use NVivo [37] Software to manage the qualitative data.

To account for correlations between observations on the same individual due to repeated measurements over time, the following quantitative analyses will be performed according to the type of variable. For the categorical dependent variables, analyses using generalized estimating equations with PROC GENMOD in SAS, which is a generalization of a traditional logistic regression, will be performed. For continuous dependent variables, Linear Mixed Models will be used with SAS’s PROC MIXED, which is a generalization of a paired data model, similar to a repeated measures ANOVA. One of the strengths of these models is that they consider the measurements of an individual, even if some data may be missing for a given period. Other procedures eliminate individuals for which a response is incomplete. If we consider the collaborative practices variable, with 24 people, 3 measurement times, and a power of 80%, we can detect an effect size of 0.3 and an intra-class coefficient of 0.82 in phase 1. For phase 2, with 33 people, 3 measurement times, and a power of 80%, an effect size of 0.4 and an intra-class coefficient of 0.76 will be detected. Power analyses will be performed using SAS version 9.4 (SAS Institute, Cary, NC).

We will apply a mixed methods approach, as it is used broadly in primary care research [38]. In each case, the qualitative and quantitative data will be collected and analysed during a similar timeframe using a convergent design method [39]. Then, the qualitative and quantitative data will be integrated [20, 40, 41] by assimilation and merged [42]. The cases will then be compared to each other to understand elements related to organizations, clinicians, trainers, patients, and the intervention itself, that have influenced the implementation and effects of the education intervention. Finally, data to document and understand scaling will be extracted, categorized, and contextualized according to scaling evaluation criteria [23].

## Discussion

This educational intervention model will allow us to better understand the complex context of PCCs [39] involving different settings and services offered [41, 43]. Also, this model will provide a better understanding of the implementation of the education intervention, the assimilation of practice guides, and its effects for each participating healthcare center. The knowledge of the following elements, presented in Table 2, will be enhanced: 1) the impact (acceptability, feasibility, adaptability, efficiency); 2) areas of study; 3) cost (resources needed); and 4) coverage (how many people trained or affected by the education intervention, deployment strategies of professional practices, deployment of intervention or strategies as expected, maintenance of intervention and strategies in settings).

**Table 2:**
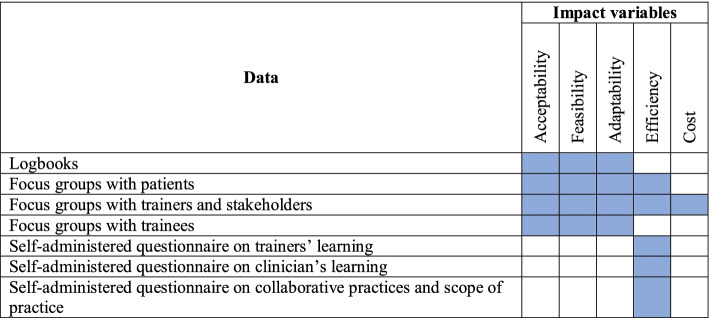
Alignment between data and impact variables

Table 2 demonstrates the alignment between what data and impact variables (acceptability, feasibility, adaptability, effectiveness, and costs).

Finally, this study protocol, based on reflective practice, patient-centered research, and focused on the needs of the community, and in collaboration with partners and patients, may serve as an evidence-based educational intervention model for further implementation in primary care.

## Data Availability

The datasets used and/or analysed during the current study are available from the corresponding author on reasonable request.

## Abbreviations

PCC: Primary care clinic
RN: Registered nurse
SW: Social worker
TTT: Train-the-trainer
